# Establishment and evaluation of four different types of patient-derived xenograft models

**DOI:** 10.1186/s12935-017-0497-4

**Published:** 2017-12-20

**Authors:** Xiaoqian Ji, Siyu Chen, Yanwu Guo, Wende Li, Xiaolong Qi, Han Yang, Sa Xiao, Guang Fang, Jinfang Hu, Chuangyu Wen, Huanliang Liu, Zhen Han, Guangxu Deng, Qingbin Yang, Xiangling Yang, Yuting Xu, Zhihong Peng, Fengping Li, Nvlue Cai, Guoxin Li, Ren Huang

**Affiliations:** 1 School of Basic Courses, Guangdong Pharmaceutical University, Guangzhou, 510006 China; 2 Guangdong Laboratory Animals Monitoring Institute, Guangdong Key Laboratory Animal Lab, 11 Fengxin Road, Science City, Guangzhou, 510663 China; 3 Department of Neurosurgery, Zhujiang Hospital, Southern Medical University, Guangzhou, 510282 China; 4 Department of General Surgery, Nanfang Hospital, Southern Medical University, 1838 Baiyun Road North, Guangzhou, 510080 China; 5 Department of Thoracic Surgery, Sun Yat-Sen University Cancer Center, Guangzhou, 510030 China; 6 Guangdong Key Laboratory for Research and Development of Natural Drug, Guangdong Medical University, Zhanjiang, 524003 Guangdong China; 7 Guangdong Provincial Key Laboratory of Colorectal and Pelvic Floor Diseases, Guangdong Institute of Gastroenterology and the Sixth Affiliated Hospital, Sun Yat-sen University, Guangzhou, 510150 China

**Keywords:** Patient derived xenograft (PDX), Glioblastoma (GBM), Lung cancer (LC), Gastric cancer (GC), Colorectal cancer (CRC)

## Abstract

**Background:**

Patient-derived xenografts (PDX) have a biologically stable in tumor architecture, drug responsiveness, mutational status and global gene-expression patterns. Numerous PDX models have been established to date, however their thorough characterization regarding the tumor formation and rates of tumor growth in the established models remains a challenging task. Our study aimed to provide more detailed information for establishing the PDX models successfully and effectively.

**Methods:**

We transplanted four different types of solid tumors from 108 Chinese patients, including 21 glioblastoma (GBM), 11 lung cancers (LC), 54 gastric cancers (GC) and 21 colorectal cancers (CRC), and took tumor tissues passaged for three successive generations. Here we report the rate of tumor formation, tumor-forming times, tumor growth curves and mortality of mice in PDX model. We also report H&E staining and immunohistochemistry for HLA-A, CD45, Ki67, GFAP, and CEA protein expression between patient cancer tissues and PDX models.

**Results:**

Tumor formation rate increased significantly in subsequent tumor generations. Also, the survival rates of GC and CRC were remarkably higher than GBM and LC. As for the time required for the formation of tumors, which reflects the tumor growth rate, indicated that tumor growth rate always increased as the generation number increased. The tumor growth curves also illustrate this law. Similarly, the survival rate of PDX mice gradually improved with the increased generation number in GC and CRC. And generally, there was more proliferation (Ki67+) in the PDX models than in the patient tumors, which was in accordance with the results of tumor growth rate. The histological findings confirm similar histological architecture and degrees of differentiation between patient cancer tissues and PDX models with statistical analysis by GraphPad Prism 5.0.

**Conclusion:**

We established four different types of PDX models successfully, and our results add to the current understanding of the establishment of PDX models and may contribute to the extension of application of different types of PDX models.

**Electronic supplementary material:**

The online version of this article (10.1186/s12935-017-0497-4) contains supplementary material, which is available to authorized users.

## Background

Although the advent of cancer cell-line culture techniques stimulated an acceleration and expansion of cancer biology discovery that continues to this day, the harsh reality is that there is a high failure rate among new oncology agents when attempting to translate preclinical efficacy into clinical benefit. The primary reasons cited for high failure rates include substantial genetic divergence between primary cancers and cell lines, and the inability of cell lines to mimic the heterogeneity of tumors in patients [1–5]. Afterwards, in vivo models based on a limited number of cancer cells previously isolated from tumors and selected prior to implantation in animals have been used extensively in tumor biology research or evaluation of anticancer drugs [6, 7]. Unfortunately, these in vivo models also fail to reproduce the tumor microenvironment and cancer cell adaptation to the innate immune system, both of which are pivotal to the architecture of the primary tumors, proliferation and metastasis [3].

One of the critical issues relevant to the failure of the preclinical models is their failure to recapitulate the heterogeneity of tumors in patients. The heterogeneity includes intratumor heterogeneity and intertumor heterogeneity, differences in the sensitivities to drug treatment, and different rates of resistance to the drugs, resulting in inadequate treatment decisions [8]. Recently, patient-derived xenograft (PDX) models of human tumor tissues obtained directly from the patients and transplanted into immune-compromised mice have gained popularity in cancer research. These models better resemble the heterogeneity of human tumors [2, 9–11]. Furthermore, PDX models have been shown to predict clinical responses to chemotherapeutic drugs more accurately than other platforms, which centralize the role of PDX models in a new generation of personalized cancer therapy [12, 13].

In February 2016, the national cancer institute (NCI) announced that at NCI-60 cell line repository, which has been used by researchers across the world for the past 25 years, would retire in late spring due to the emergence of PDX models [14]. An article in late 2015 in the international academic journal Nature medicine reported that researchers at the Novartis institute of biomedical research had successfully established about 1000 cases of patient-derived xenograft animal models. The researchers had also validated these models and proved that the clinical relevance of these models is as high as 90%. The results indicate potential applications of the PDX models in preclinical drug evaluation and prediction of precise clinical effects of drug compounds [15].

At present, the research category of PDX models is mainly focused on the following aspects: assessment of cellular, histology, epigenetic and molecular signatures of patient tumor tissue with PDX model, oncology drug development, and clinical research [2, 16–19]. However, detailed PDX modeling methods have not been widely disseminated, and the factors that affect the rates of tumor formation and mortality are also not clear. Some of the urgent questions awaiting answers on PDX models include: after transplantation, how long does it take for the tumor to grow up to 500 mm^3^, whether different types of tumors have similar tumor formation rates, how does serial passaging effect the original tumor, how to ensure stable tumor formation rate, and so on.

Hence, in the present study, we established a set of PDXs by transplanting 21 GBM, 11 LC, 54 GC and 21 CRC patient tumor specimens into highly immune-compromised NOD.Cg-*Prkdc* ^*scid*^ *Il2rg* ^*tm1Wjl*^ */SzJ* (NSG) mice, and demonstrated that these PDXs reflected the histological and biochemical characteristics of the original cancer. We also present a summary of the important factors that influence the tumor formation rate and the mortality in PDX mice. We hope to perfect and refine the methodology of establishing PDX mice model, which can be eventually used as a reference for future applications of the PDX model.

## Materials and methods

### Materials and reagents

Hanks fluid (Leagene,CC0033), glycerin (Sigma,G9012), SP Rabbit HRP Kit (DAB) was brought from Cwbiotech (CW2035S), HRP-labeled Goat Anti-Mouse IgG (H+L) was purchased from Beyotime Biotechnology (Shanghai, China), Antibodies were obtained from the following sources: anti-HLA-A (Abcam, ab52922), anti-CD45 (Abcam, ab10558), anti-Ki67 (Abcam, ab15580), anti-glial fibrillary acidic protein (Dako), anti-Human Carcinoembryonic Antigen (clone II-7, Dako).

### Patient tissue procurement

All 21 GBM, 11 LC, 54 GC, 21 CRC patients underwent surgical operations at Zhujiang Hospital of Southern Medical University (Guangzhou, China), Nanfang Hospital, Southern Medical University (Guangzhou, China), Sun Yat-Sen University Cancer Center (Guangzhou, China) and The Sixth Affiliated Hospital, Sun Yat-sen University (Guangzhou, China), respectively. All tissues were obtained intraoperatively from April 2016 to March 2017. None of the patients received any chemotherapy or radiotherapy prior to surgery. Tissue histology was confirmed by two pathologists. Prior written informed consent was obtained from all patients and the study protocol received Ethics Board approval in all hospitals. Fresh harvested tumor specimens were obtained from the edge of whole tumor tissues to maintain to minimize the necrotic parts. All tissues were transported to our laboratory in transport media (hanks liquid). The tumor specimens were divided into three parts for the following purposes: implantation into NSG mice for xenograft model establishment, snap freezing in liquid nitrogen for DNA/RNA extraction, and fixed in 4% paraformaldehyde solution for 24 h and embedded into paraffin for histopathological analyses.

### Patient tumor xenografts

The generation harboring patient-derived tumor tissue is termed as F1. And mice were bred and maintained at the local animal facility according to the legislation and ethical approval was obtained for the establishment of patient derived xenografts (PDX). We carried out the transplant on a UV ultra clean table. Before implantation, the necrotic tissues were removed, and the tissue was rinsed with sterile Hanks fluid. Solid tumor tissues were selected and cut into approximately 3 × 3 × 3 mm^3^ pieces for preparation. 6–8 week-old NSG female mice were selected for the establishment of PDX models. Recipient immunocompromised NSG mice were given general anesthesia via isoflurane inhalation continuously at 1–3% concentration with an oxygen flow rate of 9–10 cc/min. The mouse was placed ventral side down with a nose-cone to provide continuous anesthesia. The left thigh was cleaned with 75% ethanol and a small horizontal 5 mm incision was made using sterile small surgical scissors. The tip of the sterile scissors was inserted into the incision, directly over the thigh, and the scissors were opened to introduce a pocket in the subcutaneous space. One individual piece of tumor tissue was inserted into the pocket using sterile forceps. The overlying skin was held together for 3–5 s with forceps to allow adequate time for drying. Monitoring for tumor growth was done up to 8 months after transplantation for patient tumor (F0). If no tumor was palpable on animals after this period, grafting was considered unsuccessful. After outgrowth of patient tumor and reaching a size of approximately 500 mm^3^, PDX tumors were harvested and passaged to another batch of NSG mice and called F2. Tumors were typically transplanted two times consecutively (i.e. up to F3). After implantation, animals were carefully tended and observed. The length and width of the xenografts were measured once a week after tumor formation, and relative tumor volume was calculated using the formula: relative tumor volume = 0.5 × length diameter × short diameter^2^ [18]. Then the tumor volume growth curve was figured out. During the growth of the tumor, the date and number of dead mice will also be recorded to calculate the survival rate of these tumor PDX models.

### Tumor cryopreservation

After dissociation, tumor tissue not used for passaging or pathologic analysis was cryopreserved for banking and later usage. One part of the tumor was directly frozen in − 80 °C, *another part* was cut into pieces in micro tubes containing chilled 10% glycerin for 24 h, and transferred to liquid nitrogen for long-term storage.

### Clinical data collection

The diagnosis of GBM, LC, GC and CRC was confirmed by histological analysis in all cases. The following patient characteristics were collected for research study only, including: gender, age, tumor site, histological grade, differentiated degree, and TNM classification.

### Pathology

The surgical resection specimen was inspected and processed according to national and international guidelines [20]. The microscopic assessment was performed by an experienced GI-pathologist and the final diagnosis was set in accordance with the WHO classification [21]. Adenocarcinomas and squamous cell carcinomas were classified according to site of origin and tumor stage, in accordance with the TNM classification of malignant tumors [22].

### H&E staining

Tissues from all PDX models and the corresponding patient tumors were harvested and fixed in 4% Paraformaldehyde solution within 24 h after resection. Sections were dehydrated and immersed in the wax prior to paraffin embedding and cut into slices of 4 μm on a microtome. Finally, the sections were stained with hematoxylin and eosin and reviewed by a pathologist to confirm the diagnosis.

### Immunohistochemistry

Prepared tissue sections of 4 μm were de-paraffinized, followed by heat and high pressure mediated antigen retrieval with citrate buffer solution (pH 7.4). Endogenous peroxidase activity was blocked with 3% hydrogen peroxide in PBS. Non-specific staining was blocked using normal goat serum for 40 min at room temperature. Primary antibodies were diluted in normal antibody dilution buffer (Solarbio), applied on tissue sections and incubated overnight at 4 °C in a humidified chamber. Next day, biotin conjugated goat anti-rabbit IgG was used as the secondary antibody while Streptavidin-HRP applied as the third step both for 30 min at room temperature. Visualization was performed using DAB detection system. Antibodies used for immunohistochemistry were: anti-HLA-A (Abcam, 1:100), anti-CD45 (Abcam, 1:100), anti-Ki67 (Abcam, 1:100), anti-glial fibrillary acidic protein (Dako, 1:100), anti-Human Carcinoembryonic Antigen (clone II-7, Dako, 1:100).

### Statistical analysis

All experiments were performed at least in triplicates and the values are expressed as mean ± SD. Statistical differences between multiple groups of data were analyzed by one-way ANOVA with Dunnett’s multiple comparisons test (GraphPad Prism 5.0). p < 0.05 was considered statistically significant.

## Results

### Schematic outline of the generation of four different types of cancer PDX models and the growth and death of PDX model mice

**Fig. 1:**
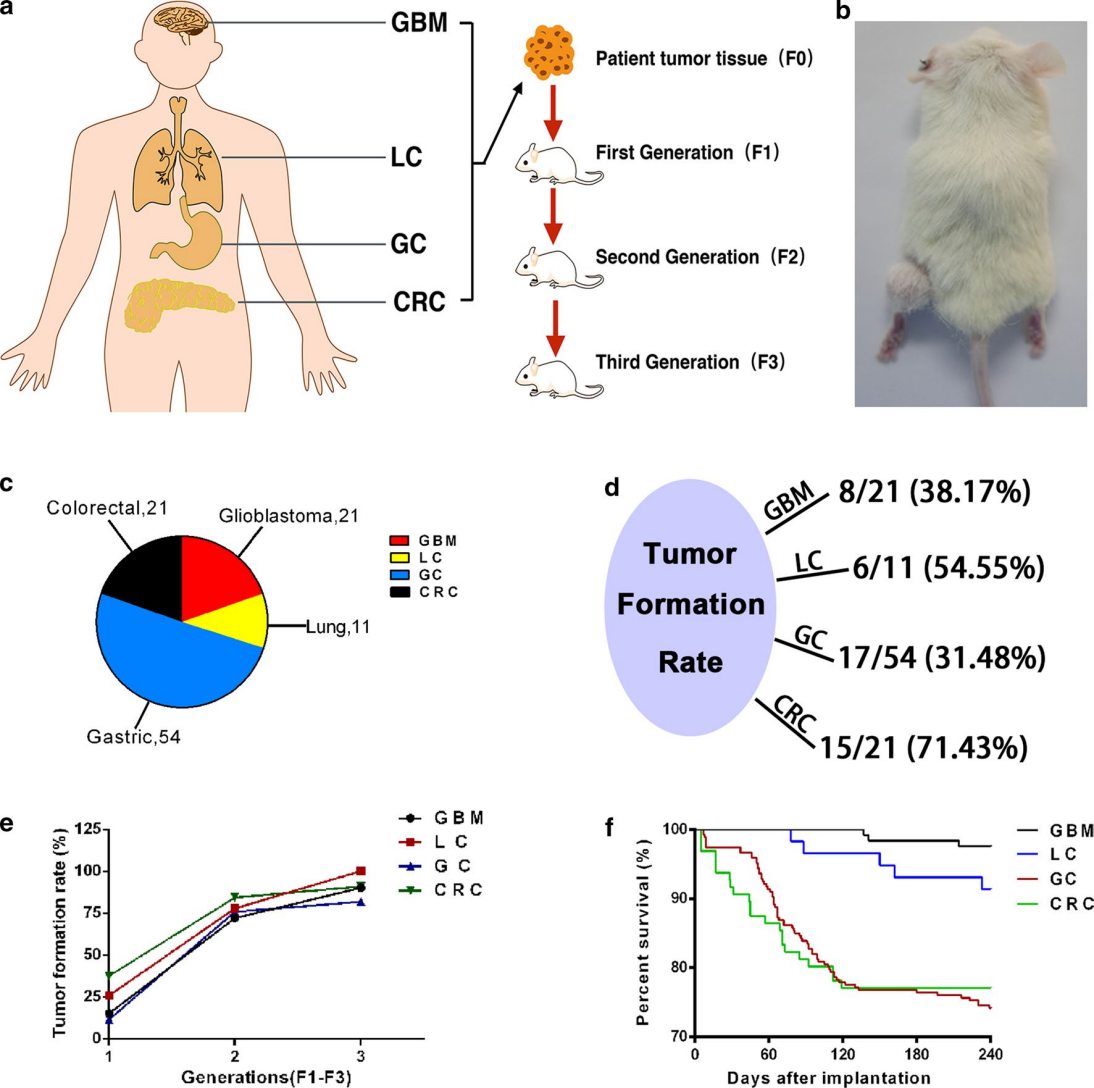
Patient-derived xenograft (PDX) mice model. **a** Schematic outline of the generation of four different cancer PDXs. **b** Tumor transplanting site. **c** The distribution of tumor cases. **d** The tumor formation rate of F0. **e** The tumor formation rate of F1–F3. **f** The survival rate of F1

We analyzed 21 GBM, 11 LC, 54 GC and 21 CRC from the hospital (Fig. 1c), and put the brief information about patients’ number, gender and WHO grade in Table 1, detailed clinical pathology reports were shown at Additional file 1: Table S1, Additional file 2: Table S2, Additional file 3: Table S3, Additional file 4: Table S4. After we got a fresh tumor specimen, one part of it was directly frozen, and the other part was used for pathological analysis, namely F0. The remainder was cut into pieces approximately 3 × 3 × 3 mm^3^, then implanted in the left thigh of the NSG mice (Fig. 1b). The rates of tumor formation in GBM, LC, GC and CRC were 8/21, 6/11, 17/54 and 15/21, respectively (Fig. 1d). The first generation mice that received patient tumor specimens were recorded as F1. In general, one patient sample was implanted into four to six mice. All GBM, LC, GC, CRC were implanted in 125, 58, 267, 96 F1 mice respectively. After transplantation, the long and short diameters of the tumor and information on the survival of the PDX mice were recorded. When the tumor volume grew to 500 mm^3^ within 8 months, PDX model was considered to be successful. The tumor formation rates of F1 were 19/125, 15/58, 31/267, 36/96 respectively (Table 2). As for survival rate of F1 mice, survival rates of GBM and LC were significantly higher than GC and CRC (Fig. 1f). As tumor volume of F1 mice grow to 500 mm^3^, the tumors were harvested and a part of these tumors was frozen while another part was used for pathological analysis. The third part was divided into sections approximately 3 × 3 × 3 mm^3^ and transplanted to the recipient mice named F2. All F1 GBM, LC, GC, CRC were implanted into 25, 9, 33, 19 F2. For F2, as in F1, the tumor growth rates and the animal survival rates were recorded. As for F1, when the tumor volume grew to 500 mm^3^ within 6 months, it was considered to be successful, the tumor formation rates of F2 were calculated as 18/25, 7/9, 25/33, and 16/19 (Table 2). And so on, the third generation was called F3, GBM, LC, GC, CRC each transplanted 10, 4, 22 and 11 mice. Likewise, make a record of growth and death of PDX model mice. Transplantation was considered successful if the tumor volume grew to 500 mm^3^ within 4 months. The tumor formation rates of F3 were 9/10, 4/4, 18/22, 10/11 (Table 2). When the tumor volume of F3 grew to 500 mm^3^, the tumors were harvest, part for frozen, part for pathological analysis. The entire process is indicated in Fig. 1a. We found that the tumor formation rate is positively correlated with generations (Fig. 1e).

**Table 1:** Clinical characteristics of biobank patients

	n		n
GBM, n = 21		GC n = 54	
Gender		Gender	
Male	10	Male	39
Female	11	Female	15
Stage		Stage	
I	4	I	6
II	6	II	15
II–III	3	III	31
III	4	IV	2
IV	4	Grafting outcome	
Grafting outcome		Successful	17
Successful	7	Unsuccessful	37
Unsuccessful	14	Success rate	17/54
Success rate	7/21		

	n		n
LC n = 11		CRC n = 21	
Gender		Gender	
Male	8	Male	12
Female	3	Female	9
Grafting outcome		Grafting outcome	
Successful	6	Successful	13
Unsuccessful	5	Unsuccessful	8
Success rate	6/11	Success rate	13/21

**Table 2:** The tumor formation rate and mortality rate of PDX models

	Tumor formation rate			Mortality rate		
	F1	F2	F3	F1	F2	F3
GBM	19/125 (15%)	18/25 (72%)	9/10 (90%)	3/125 (2%)	1/25 (4%)	0/10 (0%)
LC	15/58 (26%)	7/9 (78%)	4/4 (100%)	3/58 (5%)	2/9 (22%)	0/4 (0%)
GC	31/267 (12%)	25/33 (76%)	18/22 (82%)	67/267 (26%)	1/33 (3%)	0/22 (0%)
CRC	36/96 (38%)	16/19 (84%)	10/11 (91%)	22/96 (23%)	1/19 (5%)	0/11 (0%)

### Comparison of the tumor growth rates in F1–F3 in each type of tumor PDX model

**Fig. 2:**
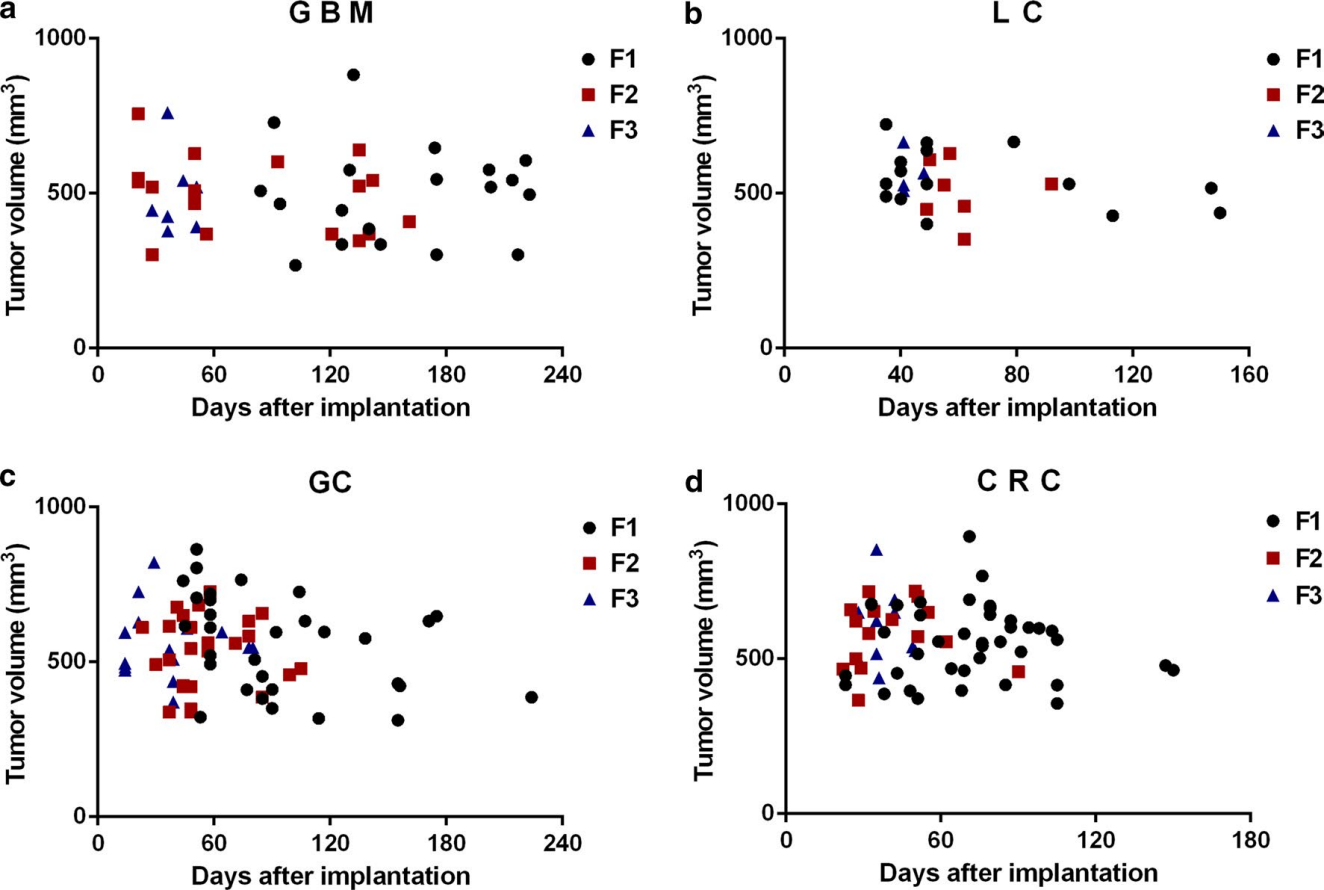
Time of tumor growth. **a** Time of GBM growth. **b** Time of LC growth. **c** Time of GC growth. **d** Time of CRC growth

19 mice implanted with GBM in the F1 stage were positive for successful tumor growth and it took 84–223 days. 18 mice in F2 grew tumor successfully, it took 22–161 days. 9 mice in F3 grew tumor successfully, it took 28–51 days (Fig. 2a). 15 mice in LC F1 grew tumor successfully, it took 35–150 days. 7 mice in F2 grew tumor successfully, it took 49–92 days. 4 mice in F3 grew tumor successfully, it took 41–48 days (Fig. 2b). 31 mice in GC F1 grew tumor successfully, it took 44–224 days, F2 had 25 mice grew tumor successfully, it took 23–105 days, 18 mice in F3 grew tumor successfully, it took 14–80 days (Fig. 2c). 36 mice in CRC F1 grew tumor successfully, it took 23–150 days. 16 mice in F2 grew tumor successfully, it took 22–90 days. 10 mice in F3 grew tumor successfully, it took 28–50 days (Fig. 2d). In brief, the longest tumor formation day of the four different tumors in F1 is 224, F2 is 161, and F3 is 80, in other word, the tumor growth rate accelerated with the increase of generation.

### Histopathological comparison of patient tissue with transplanted tumors and the tumor volume growth curves for all generations in GBM-16

**Fig. 3:**
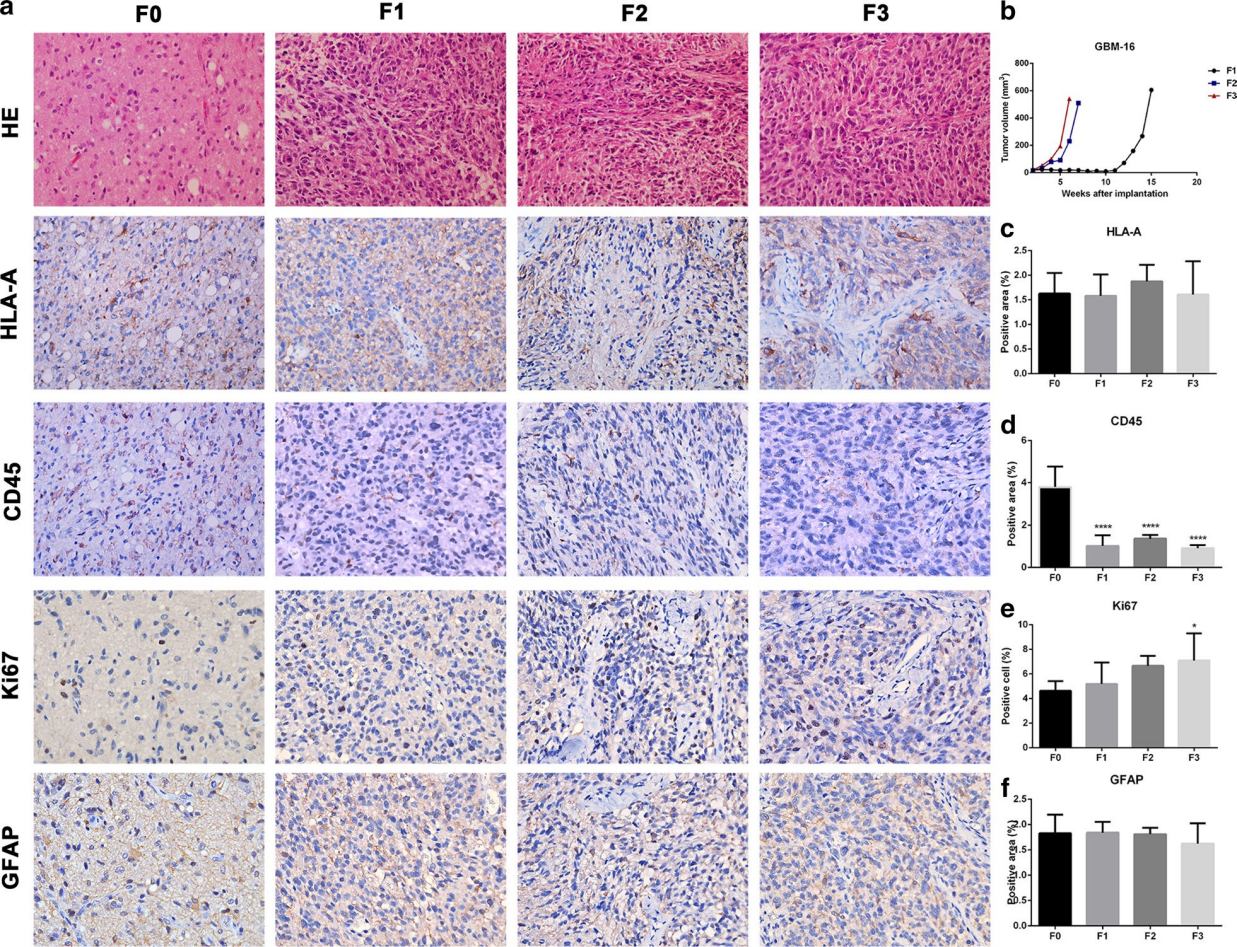
Histopathological comparison of patient tissue with transplanted tumors and the tumor growth curves of GBM-16. **a** H&E and immunohistochemistry staining of HLA-A, CD45, Ki67, GFAP on (F0) patient tumor and derived (F1–F3) transplanted tumors. **b** The tumor growth curve of GBM-16. **c**, **d**, **f** The positive area of HLA-A, CD45 and GFAP was quantified. **e** Quantification of cells positive for Ki67. *p < 0.05, ****p < 0.0001 vs F0

In F1, F2 and F3, some tumor cells were similar to F0, the tissue heteromorphism was more obvious than F0, and the tumor cells showed a dense distribution (Fig. 3a). Tumor volume growth curves for GBM-16 in F2 and F3 were visibly faster than F1 (Fig. 3b). As for the immunohistochemical data, HLA-A and GFAP protein expression in all generations were similar (Fig. 3c, f). CD45 protein expression in F1, F2 and F3 was dramatically reduced in comparison to the expression levels in F0 (Fig. 3d). The numbers of Ki67 positive cells in F1 and F2 were no less than F0, but F3 showed higher numbers of Ki67 positive cells (Fig. 3e).

### Histopathological comparison of patient tissues with transplanted tumors and the tumor volume growth curves of all generations in LC-9

**Fig. 4:**
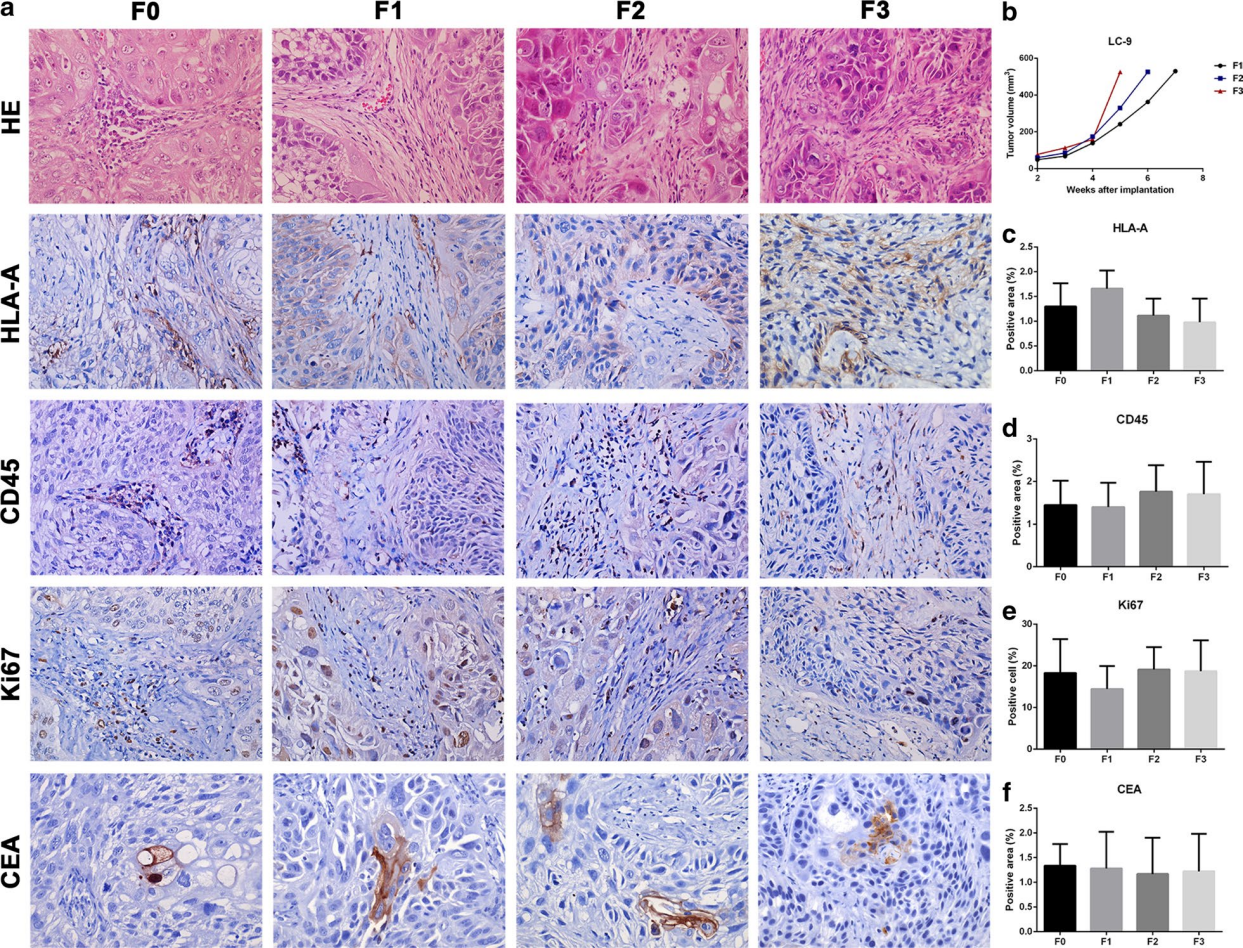
Histopathological comparison of patient tissues with transplanted tumors and the tumor growth curves of LC-9. **a** H&E and immunohistochemistry staining of HLA-A, CD45, Ki67, CEA on (F0) patient tumor and derived (F1-F3) transplanted tumors. **b** The tumor growth curve of LC-9. **c**, **d**, **f** Areas positive for HLA-A, CD45, CEA were quantified. **e** Quantification of cells positive for Ki67

Compared with F0, tumor cells in F1, F2 and F3 had obvious heteromorphism and pathologic mitosis. Besides, some tumor cells were similar to F0, and they all owned similar tissue stroma (Fig. 4a). Tumor growth rates of LC-9 were accelerated as the generation number increased (Fig. 4b). HLA-A, CD45, CEA protein expression and cells positive for Ki67 in F1, F2 and F3 were similar to F0 (Fig. 4c–f).

### Histopathological comparison of patient tissue with transplanted tumors and the tumor volume growth curves for all generations in GC-28

**Fig. 5:**
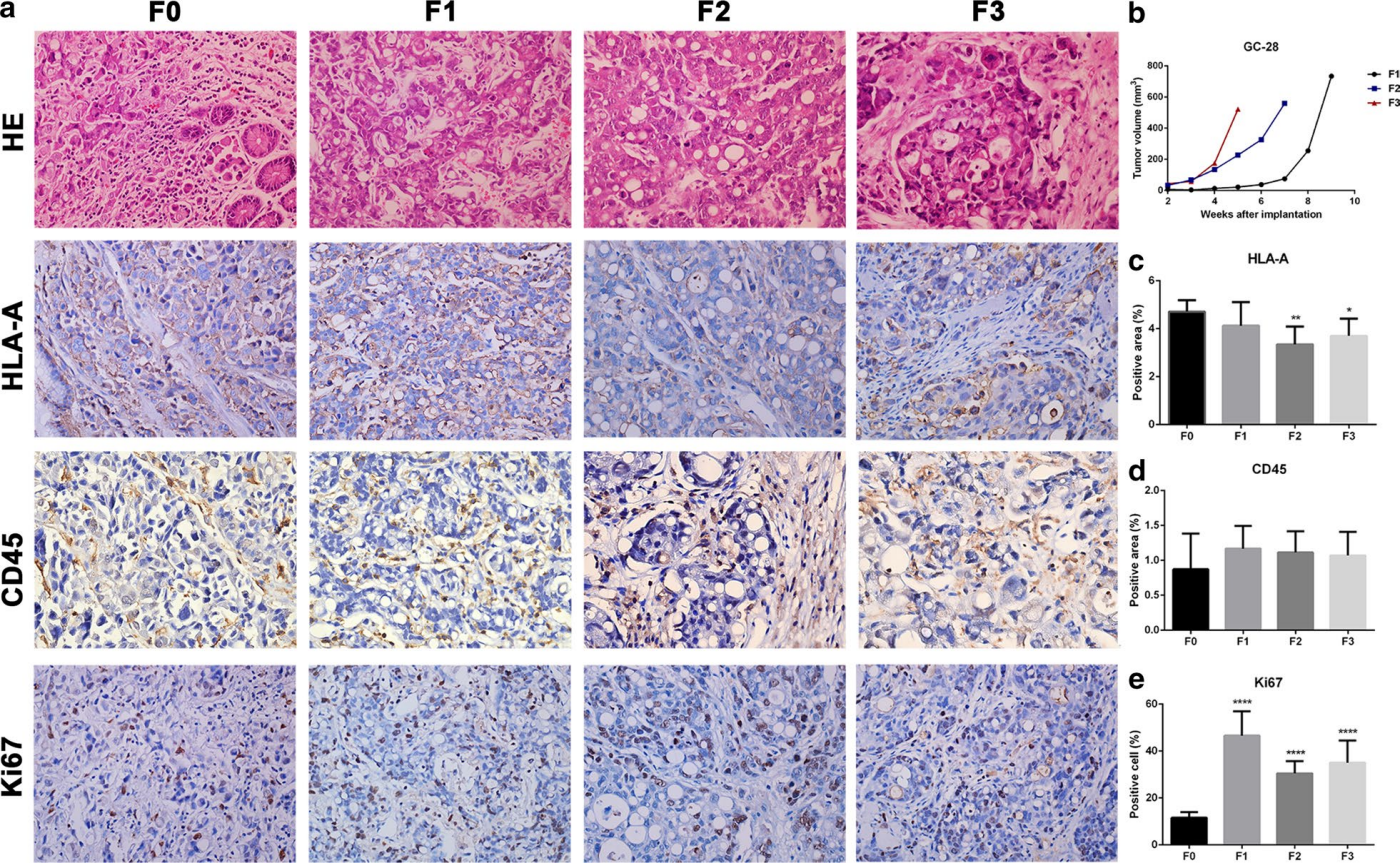
Histopathological comparison of patient tissue with transplanted tumor tissues and the tumor growth curves of GC-28. **a** H&E and immunohistochemistry staining for HLA-A, CD45, Ki67 on (F0) patient tumor and derived (F1-F3) transplanted tumors. **b** The tumor growth curves of GC-28. **c**, **d** The areas positive for HLA-A, CD45 was quantified. **e** Cells positive for Ki67 were quantified. *p < 0.05, **p < 0.01, ****p < 0.0001 vs F0

Inflammatory reactions in F1, F2, and F3 were lesser than the reactions observed in F0, while the tumor cell density was higher. However, tumor tissues and cells in all generations showed obvious heteromorphism and similar morphology (Fig. 5a). Accelerated tumor growth rate was observed for GC-28 as the generation increased (Fig. 5b). HLA-A protein expression in F1 was the same as F0, but decreased in F2 and F3 (Fig. 5c). CD45 protein expression in F1, F2 and F3 were similar to F0 (Fig. 5d). More cells were positive for Ki67 in F1, F2, and F3 as compared to the F0 (Fig. 5e).

### Histopathological comparison of patient tissues with transplanted tumors and the tumor volume growth curves for all generations in CRC-12

**Fig. 6:**
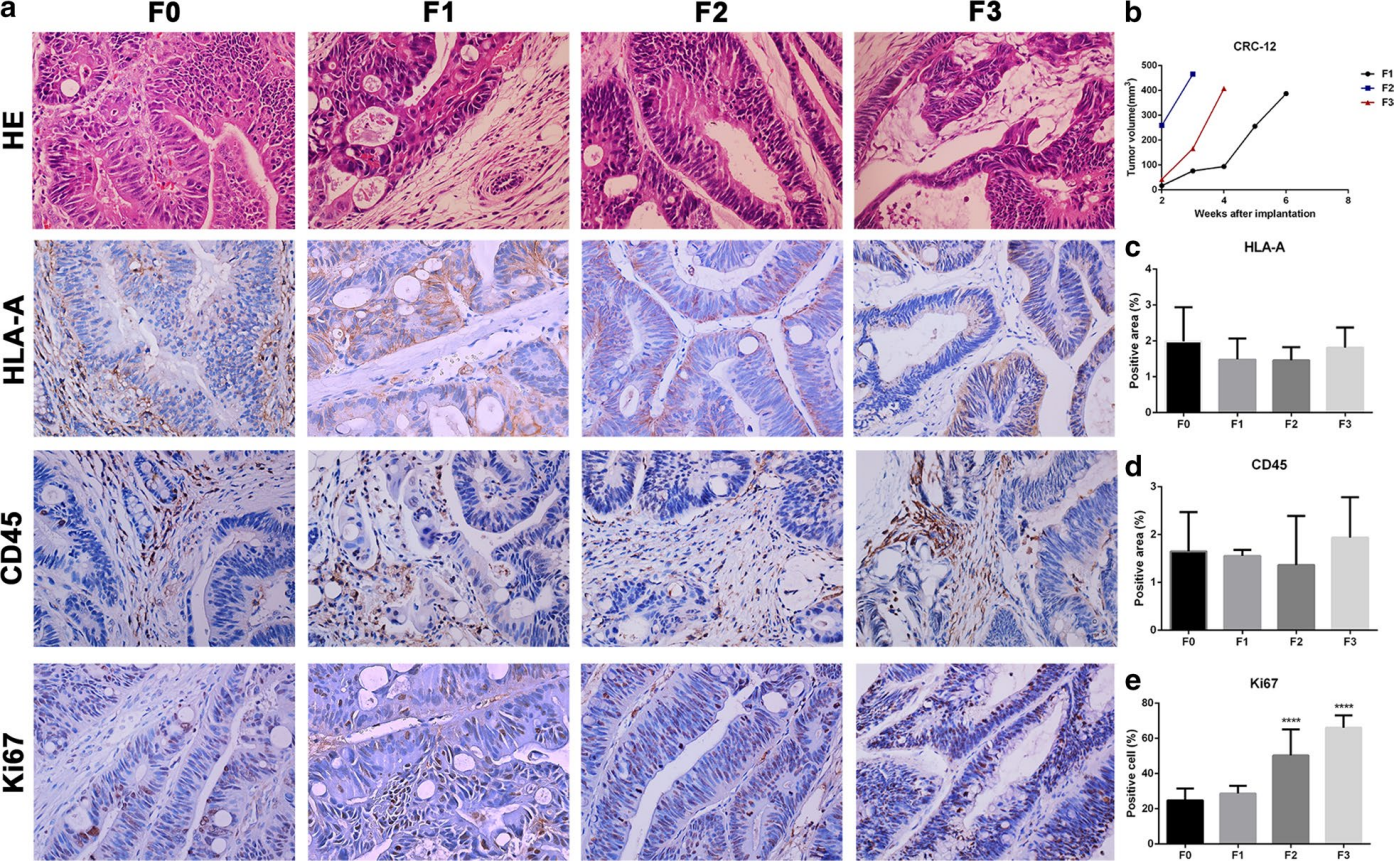
Histopathological comparison of patient tissue with transplanted tumors and the tumor growth curves of CRC-12. **a** H&E and immunohistochemistry staining for HLA-A, CD45, Ki67 in patient tumor (F0) and derived (F1–F3) transplanted tumors. **b** Tumor growth curve of CRC-12. **c**, **d** HLA-A positive areas and, CD45 positive areas were quantified. **e** Cells positive for Ki67 were quantified. ****p < 0.0001 vs F0

According to the H&E staining, xenograft models maintained the tumor tissue cell morphology and the mesenchymal components of parental tumor tissue (Fig. 6a). The tumor growth rate for CRC-12 in F2 and F3 were faster than F1. However, F3 was slower than that of F2 (Fig. 6b). HLA-A and CD45 protein expression in F1, F2 and F3 were same as F0 (Fig. 6c, d). Cells positive for Ki67 were similar in F1 and F0 while the number of cells positive increased in F2 and F3 compared to F1 (Fig. 6e).

## Discussion

Malignant tumors pose a serious threat to human health and the quality of life. Researchers have been working to develop a model for human tumors that mimics the tumor microenvironment and can be used to understand the impact of various anti-cancer treatments. Preclinical trials for validation of potential therapeutic targets via in vitro and in vivo models are regarded indispensable in the development of anti-cancer drug therapeutics [23]. However, standard tumor cell lines expanded in vitro and cell line-derived xenografts show low predictability of drug sensitivities because of failure to accurately reflect the genetic and functional aspects related to the heterogeneity of the tumor cells [24]. In contrast, animal models are regarded as appropriate tools to resolve both basic and clinical research problems [25]. Therefore, patient-derived xenografts (PDX) can overcome the limitations of in vitro models by faithfully recapitulating the histological and functional heterogeneity observed in primary tumor samples [2].

In recent years, patient derived xenografts (PDXs) have been used to evaluate targeted treatments for different types of tumors, such as breast and non-small cell lung cancer, esophageal squamous cell carcinoma. The response of PDXs to chemotherapy has been shown to resemble the patient response in different clinical treatment trials [16, 26, 27]. In the last decade, the PDX model has been widely used for preclinical research. However, few studies have detailed the methodology of many types of PDX establishment and various factors that affect the tumor formation rate. Therefore, we summarized the detailed records during the development of the PDX models for different human tumors to provide a reliable reference.

To maximize the utility of a PDX model database in determining the treatment options for patients, serial passaging is crucial to expand primary tumor tissue for biobanks and cohorts for preclinical mouse avatar trials. In general, all PDXs eventually lose the human stromal elements and therefore, the consensus is that a low-passage number is ideal to conserve the histological and genetic integrity of the primary tumor [28]. In this paper, we passaged the parental tumors (F0) up to three generations-F1, F2, and F3 respectively.

In the past, generally all PDX models are implanted with the patient tumor tissue in the flank of mice. We however implanted the tumor in the left thigh of mice for three reasons: it is convenient to observe and measure the tumor size, fixed position of tumor was in favor of fixed point chemotherapy for our future studies and it is easy to determine whether the tumor is metastatic, following tumor resection. Most important of all, we found that there are many related reports in the very early research injecting cell line or solid tumor subcutaneously into the right thigh or hind limb of mice and calculating tumor volume to benefit fixed point radiotherapy [29–33].

To evaluate whether established PDX models resemble the original patient tumor, the histology of tumor samples from the PDX was assessed based on representative hematoxylin–eosin (HE) staining of the parental tumor (F0) and homologous F1, F2, F3 tumors. We found that these xenografts closely rebuilt the original patient tumors and the tumor cells had similar morphologies and/or associated intercellular stromal elements in successive PDX generations, thereby underlining the value of PDX for modeling morphologically heterogeneous patient tumors in vivo. Although we did not examine the gene expression patterns in this paper, we confirmed that the xenograft tumors-F1, F2 and F3 exhibited similar immunohistochemical phenotypes to that of patient’s original tumor, which indicates that PDXs primarily maintain the histopathological and molecular characteristics of the parental tumor.

In addition, we found certain patterns during serial passaging in vivo. Obviously, tumor formation rate increased significantly in subsequent tumor generations. Also, the survival rates of GC and CRC were remarkably higher than GBM and LC. As for the time required for the formation of tumors, which reflects the tumor growth rate, indicated that tumor growth rate always increased as the generation number increased. The tumor growth curves also illustrate this law. Similarly, the survival rate of PDX mice gradually improved with the increased generation number in GC and CRC. And generally, there was more proliferation (Ki67+) in the PDX models than in the patient tumors, which was in accordance with the results of tumor growth rate.

## Conclusion

In summary, we established four different types of PDX models successfully, and our findings are of particular relevance for current and future preclinical mouse studies correlating drug screening and personalized anti-tumor therapy in different types of tumors, including GBM, LC, GC and CRC. It is quite clear that further large scale studies are required to validate our conclusion, especially for LC. In addition, we are planning to investigate chemotherapeutic drugs in PDX mice models. Nonetheless, future studies are still warranted to determine if PDX technique can be widely used as an important tool to develop the novel drugs against drug-resistant tumors in preclinical trials and to guide in the treatment decisions for individual patients upon tumor progression on standard treatment.

## Additional files

**Additional file 1: Table S1.** Clinical characteristics of GBM patients.

**Additional file 2: Table S2.** Clinical and pathological characteristics of LC patients.

**Additional file 3: Table S3.** Clinical characteristics of GC patients.

**Additional file 4: Table S4.** Clinical and pathological characteristics of CRC patients.

## Abbreviations

PDX: patient derived xenograft
GBM: glioblastoma
LC: lung cancer
GC: gastric cancer
CRC: colorectal cancer
GFAP: glial fibrillary acidic protein
CEA: carcinoembryonic antigen
F0: the patient tumor
F1: first generation
F2: second generation
F3: third generation
