# Supplementary material for: Establishment and evaluation of four different types of patient-derived xenograft models
Source: Cancer Cell Int. 2017 Dec 20;17:122. doi: 10.1186/s12935-017-0497-4 (PMC5738885; doi:10.1186/s12935-017-0497-4)
Supplement: Supplementary file 1 — Additional file 1: Table S1. Clinical characteristics of GBM patients. [file 12935_2017_497_MOESM1_ESM.docx]

| **Table S1** Clinical characteristics of GBM patients | | | | |
| --- | --- | --- | --- | --- |
| ID | Gender | Age | Stage (WHO) | Grafting outcome |
| GBM08 | M | 76 | Ⅱ | unsuccessful |
| GBM13 | M | 45 | Ⅱ | unsuccessful |
| GBM14 | F | 28 | Ⅳ | unsuccessful |
| GBM16 | F | 54 | Ⅱ-Ⅲ | successful |
| GBM17 | F | 49 | Ⅰ | successful |
| GBM18 | F | 43 | Ⅲ | unsuccessful |
| GBM19 | M | 30 | Ⅳ | successful |
| GBM20 | F | 59 | Ⅲ | unsuccessful |
| GBM21 | F | 67 | Ⅱ-Ⅲ | successful |
| GBM22 | M | 23 | Ⅲ | unsuccessful |
| GBM23 | F | 41 | Ⅳ | successful |
| GBM24 | M | 60 | Ⅱ | unsuccessful |
| GBM25 | F | 52 | Ⅱ | unsuccessful |
| GBM26 | M | 41 | Ⅱ | unsuccessful |
| GBM27 | M | 53 | Ⅰ | unsuccessful |
| GBM28 | M | 66 | Ⅱ-Ⅲ | unsuccessful |
| GBM31 | M | 62 | Ⅰ | successful |
| GBM34 | M | 9 | Ⅰ | unsuccessful |
| GBM35 | F | 57 | Ⅲ | successful |
| GBM36 | F | 37 | Ⅱ | successful |
| GBM38 | F | 45 | Ⅳ | unsuccessful |
