# Supplementary material for: Establishment and evaluation of four different types of patient-derived xenograft models
Source: Cancer Cell Int. 2017 Dec 20;17:122. doi: 10.1186/s12935-017-0497-4 (PMC5738885; doi:10.1186/s12935-017-0497-4)
Supplement: Supplementary file 2 — Additional file 2: Table S2. Clinical and pathological characteristics of LC patients. [file 12935_2017_497_MOESM2_ESM.docx]

| **Table S2** Clinical and pathological characteristics of LC patients | | | | |
| --- | --- | --- | --- | --- |
| ID | Gender | Diagnosis | Tumor differentiation | Grafting outcome |
| LC01 | M | bronchioloalveolar carcinoma | well | successful |
| LC02 | F | Acinar Adenocarcinoma | poor | successful |
| LC03 | M | basal cell carcinoma | poor-moderate | unsuccessful |
| LC04 | M | basal cell carcinoma | poor-moderate | successful |
| LC05 | F | bronchioloalveolar carcinoma | well | unsuccessful |
| LC06 | M | bronchioloalveolar carcinoma | well | unsuccessful |
| LC07 | M | papillary adenocarcinoma | moderate-well | successful |
| LC08 | M | bronchioloalveolar carcinoma | moderate | successful |
| LC09 | F | Large cell carcinoma | na | successful |
| LC10 | M | Acinar Adenocarcinoma | moderate | unsuccessful |
| LC11 | M | bronchioloalveolar carcinoma | well | unsuccessful |
