# Supplementary material for: Establishment and evaluation of four different types of patient-derived xenograft models
Source: Cancer Cell Int. 2017 Dec 20;17:122. doi: 10.1186/s12935-017-0497-4 (PMC5738885; doi:10.1186/s12935-017-0497-4)
Supplement: Supplementary file 3 — Additional file 3: Table S3. Clinical characteristics of GC patients. [file 12935_2017_497_MOESM3_ESM.docx]

| **Table S3** Clinical characteristics of GC patients | | | |
| --- | --- | --- | --- |
| GC n=54 | | | |
| Gender | n | Stage (T) | n |
| male | 39 | T1a | 1 |
| female | 15 | T1b | 4 |
| Tumor location | n | T2 | 2 |
| U | 20 | T3 | 17 |
| M | 10 | T4 | 30 |
| L | 24 | Stage (N) | n |
| Tumor differentiation | n | N0 | 13 |
| well | 3 | N1 | 10 |
| moderate-well | 1 | N2 | 11 |
| moderate | 6 | N3 | 19 |
| poo-well | 1 | Nx | 1 |
| poor-moderate | 7 | Stage (M) | n |
| poor | 34 | M0 | 52 |
| na | 2 | MI | 2 |
