# Supplementary material for: Establishment and evaluation of four different types of patient-derived xenograft models
Source: Cancer Cell Int. 2017 Dec 20;17:122. doi: 10.1186/s12935-017-0497-4 (PMC5738885; doi:10.1186/s12935-017-0497-4)
Supplement: Supplementary file 4 — Additional file 4: Table S4. Clinical and pathological characteristics of CRC patients. [file 12935_2017_497_MOESM4_ESM.docx]

| **Table S4** Clinical and pathological characteristics of CRC patients | | | | |
| --- | --- | --- | --- | --- |
| ID | Gender | Stage (TNM) | Diagnosis | Tumor differentiation |
| CRC01 | F | na | adenocarcinoma of the colon | moderate |
| CRC02 | F | T4N4 | carcinoma of sigmoid | moderate |
| CRC03 | F | pT3N1M0 | anorectal adenocarcinoma of the rectum | na |
| CRC04 | F | pT1N0M0 | carcinoma of sigmoid | na |
| CRC05 | M | T3N1M0 | carcinoma of sigmoid | moderate |
| CRC06 | F | pT4aN0M0 | Colon hepatic carcinoma | na |
| CRC08 | M | T3 | carcinoma of sigmoid | moderate |
| CRC09 | M | pT4bN1aM0 | Rectal squamous cell carcinoma | na |
| CRC11 | M | pT4aN0M0 | Colon hepatic carcinoma | na |
| CRC12 | F | na | adenocarcinoma of the colon | moderate |
| CRC13 | M | pT3N1M0 | carcinoma of sigmoid | na |
| CRC14 | F | na | carcinoma of sigmoid | moderate |
| CRC16 | M | T4bN2b | lower rectal cancer | na |
| CRC17 | F | pT3N1M0 | carcinoma of sigmoid | na |
| CRC18 | M | T3N0M0 | adenocarcinoma of rectum | moderate |
| CRC20 | M | T4aN1M0 | Ascending colon adenocarcinoma | moderate |
| CRC22 | M | T4aN2b | carcinoma of the rectum | na |
| CRC23 | F | pT2N0M0 | Transverse colon adenocarcinoma | moderate |
| CRC25 | F | T3 | carcinoma of sigmoid | na |
| CRC26 | M | T3N2bM0 | adenocarcinoma of rectum | moderate |
| CRC27 | M | T3N1M0 | carcinoma of sigmoid | na |
